# Does a Screening Trial for Spinal Cord Stimulation in Patients With Chronic Pain of Neuropathic Origin Have Clinical Utility (TRIAL-STIM)? 36-Month Results From a Randomized Controlled Trial

**DOI:** 10.1227/neu.0000000000002165

**Published:** 2022-10-13

**Authors:** Sam Eldabe, Sarah Nevitt, Sara Griffiths, Ashish Gulve, Simon Thomson, Ganesan Baranidharan, Rachel Houten, Morag Brookes, Anu Kansal, Jenny Earle, Jill Bell, Rod S. Taylor, Rui V. Duarte

**Affiliations:** * Department of Pain Medicine, The James Cook University Hospital, Middlesbrough, UK;; ‡ Liverpool Reviews and Implementation Group, University of Liverpool, Liverpool, UK;; § Department of Pain Medicine and Neuromodulation, Mid and South Essex University Hospitals, Essex,UK;; ‖ Leeds Neuromodulation Centre, Leeds Teaching Hospitals, Leeds, UK;; ¶ Patient and Public Involvement Representatives, Middlesbrough, UK;; # College of Medicine and Health, University of Exeter, Exeter, UK;; ** MRC/CSO Social and Public Health Sciences Unit & Robertson Centre for Biostatistics, Institute of Health and Well Being, University of Glasgow, Glasgow, UK

**Keywords:** Randomized controlled trial, Screening trial, Spinal cord stimulation, Neuropathic pain, Long-term follow-up

## Abstract

Screening trials before full implantation of a spinal cord stimulation device are recommended by clinical guidelines and regulators, although there is limited evidence for their use. The TRIAL-STIM study showed that a screening trial strategy does not provide superior patient pain outcome at 6-month follow-up compared with not doing a screening trial and that it was not cost-effective.

**OBJECTIVE:**

To report the long-term follow-up results of the TRIAL-STIM study.

**METHODS:**

The primary outcome of this pragmatic randomized controlled trial was pain intensity as measured on a numerical rating scale (NRS) and secondary outcomes were the proportion of patients achieving at least 50% and 30% pain relief at 6 months, health-related quality of life, and complication rates.

**RESULTS:**

Thirty patients allocated to the “Trial Group” (TG) and 36 patients allocated to the “No Trial Group” (NTG) completed outcome assessment at 36-month follow-up. Although there was a reduction in NRS pain and improvements in utility scores from baseline to 36 months in both groups, there was no difference in the primary outcome of pain intensity NRS between TG and NTG (adjusted mean difference: −0.60, 95% CI: −1.83 to 0.63), EuroQol-5 Dimension utility values (adjusted mean difference: −0.02, 95% CI: −0.13 to 0.10), or proportion of pain responders (33% TG vs 31% NTG). No differences were observed between the groups for the likelihood of spinal cord stimulation device explant or reporting an adverse advent up to 36-month follow-up.

**CONCLUSION:**

The long-term results show no patient outcome benefit in undertaking an SCS screening trial.

**ABBREVIATIONS:**
AE: adverse event
CONSORT: Consolidated Standards of Reporting Trials
EQ-5D: EuroQol-5 Dimension
EQ-VAS: EuroQol Visual Analog Scale
FDA: Food and Drug Administration
IPG: implantable pulse generator
MCID: minimal clinical important difference
MD: mean difference
NRS: numerical rating scale
NTG: no trial group
PSPS-T2: persistent spinal pain syndrome type 2
RCT: randomized controlled trial
SCS: spinal cord stimulation
TG: trial group.

The use of spinal cord stimulation (SCS) for inhibition of pain was first described in 1967.^1^ A number of randomized controlled trials (RCTs) and economic evaluations support the clinical and cost-effectiveness of SCS for neuropathic pain.^2-15^

Despite many advances in SCS waveforms,^16-18^ target neural tissue,^19,20^ and feedback technology,^6^ common to all RCTs to date is that patients are required to undergo a screening trial to evaluate early response to therapy before full implantation of the SCS device. Screening trials are recommended by clinical guidelines and regulators including the Food and Drug Administration (FDA) in the United States.^21-24^ Such a screening trial allows patients to experience the sensation generated by SCS and potential pain relief to be achieved. A successful trial has been defined as the patient reporting ≥50% pain relief with stable or reduced pain medications and with stable levels of daily activity.^23^

Although a successful screening trial should help to identify those patients who would most benefit from SCS and obtain long-term pain relief, there are drawbacks to such a screening trial strategy. Duplication of a clinical procedure at screening and full implant increase patient exposure to infection (because of bacterial colonization of the lead skin exit site). Higher infection rates have been reported for 28-day trials compared with screening trials with shorter durations.^25,26^

Furthermore, the results from the TRIAL-STIM RCT showed there was no evidence that a screening trial provides superior patient outcomes or is cost-effective compared with not doing a screening trial.^27^ Qualitative results of the TRIAL-STIM also indicated that the patients were not supportive of the concept of a trial.^28^

An important limitation of the TRIAL-STIM RCT results was that follow-up was limited to 6 months and therefore too short to evaluate long-term response to SCS. The aim of this report is, therefore, to report the long-term results of the TRIAL-STIM trial.

## METHODS

### Study Design

TRIAL-STIM was a multicenter, single-blind, parallel 2 group randomized trial (ISRCTN, ISRCTN60778781). The study was conducted across 3 participating sites in the United Kingdom: South Tees Hospitals NHS Foundation Trust (The James Cook University Hospital), Basildon and Thurrock University Hospitals NHS Foundation Trust, and Leeds Teaching Hospitals NHS Trust. The full study protocol has been published previously.^29^ The study was approved by the UK Health Research Authority North East Research Ethics Committee (17/NE/0056). The trial was conducted and reported in accordance with Consolidated Standards of Reporting Trials (CONSORT) guidelines.^30^

### Study Participants

Participants were eligible for SCS if they met the following criteria according to NHS guidance (NICE TA159)^21^: neuropathic pain of intensity of at least 5 on a numerical rating scale (NRS); had endured pain for longer than 6 months despite receiving suitable conservative medical and surgical management; had undergone a satisfactory multidisciplinary assessment by a team with experience in providing SCS; and had the capacity to provide informed consent. Full eligibility criteria have been previously reported.^27,29^

### Randomization and Masking

Participants were allocated (1:1) to 1 of 2 groups: trial group (TG) with a screening trial followed by SCS implantation in light of the screening trial result, or no trial group (NTG) with a strategy of SCS implantation only. Patients were randomized to groups through a password-protected web-based system developed and maintained by the Exeter Clinical Trials Unit. The allocation was stratified by center, and minimized on patient age (≥65 or <65 years), sex, and presence of persistent spinal pain syndrome type 2 (PSPS‐T2). The methods for randomization and masking have been previously described.^27,29^

### Procedures

Type of device and stimulation were not restricted, and devices from 4 major SCS manufacturers were used.

### Screening Trial and Implantation Strategy (TG)

Patients who were randomly assigned to the TG arm underwent a screening trial that involved passing an external or an internalized tunneled SCS lead, or leads, to an external stimulator in accordance with the center's standard operating procedure.^29^ Considering the RCTs^3,31^ listed in the NICE TA159^21^ clinical evidence section along with international guidelines,^23^ a screening trial was defined as successful when a patient obtained ≥50% pain relief and satisfactory on table paresthesia coverage (≥80%) of the pain area, observed a reduction in pain medications or reported improvement in quality of life and function, and successful location of leads at anatomic target for paresthesia-free therapies. Patients who did not have a successful screening trial did not receive an implant but all patients were followed up. Patients who had a successful trial had the implantation of the implantable pulse generator (IPG) on a separate occasion.

### Implantation-Only Strategy (NTG)

In the NTG group, all patients received a permanent implant in one surgery when the following was observed: satisfactory on table paresthesia coverage (≥80%) of the pain area, no dislike of sensations,^32^ and satisfactory anatomic lead location for paresthesia-free devices.

### Outcomes

The primary outcome was pain intensity assessed on the NRS.^33^ Secondary outcomes assessed at 36-month follow-up were the proportion of patients achieving at least 50% and 30% pain relief as measured on the NRS,^33^ health-related quality of life using the EuroQol-5 Dimension (EQ-5D)-5L tool,^34^ and complication rates.

### Statistical Analysis

The sample size calculation has been described elsewhere.^27,29^ The mean time to follow-up was 37.3 (range 36-40 months) and 37.1 (range 36-40 months) for the TG and NTG groups, respectively. The follow-up time is subsequently referred to as 36-month follow-up.

The primary analysis compared primary and secondary outcomes between randomized groups including patients who completed the 36-month follow-up assessment.^27^ No imputation of missing data is conducted for the analyses at 36 months to avoid making assumptions over long-term follow-up periods. A complete case assessment was considered to be more appropriate than conducting an intention-to-treat analysis with or without data imputation at 36 months to avoid making assumptions about missing data over long-term follow-up periods.

Linear regression methods with adjustment for baseline outcome scores and stratification/minimization variables were used to compare continuous outcomes. Logistic regression analysis adjusting for stratification/minimization variables were used to compare binary outcomes.

All analyses were performed using STATA v14.0 (StataCorp LP).

## RESULTS

From June 2017 to September 2018, 137 patients were assessed for eligibility, with 105 patients proceeding to enrolment. Of the 105 participants, 54 were randomized to the screening TG. Seven of the patients in TG withdrew before the screening trial; of the remaining 47, an unsuccessful screening trial was observed for 5 (11%) patients and 42 (89%) patients had a successful screening trial and were implanted with an SCS system. Forty-nine of the 51 NTG patients received an SCS implant. Thirty TG patients and 36 NTG patients completed the follow-up at 36 months (Figure). Loss to follow-up in all instances was due to failure to reach the patients via telephone (maximum of 3 attempts), email, and letter.

**FIGURE.**
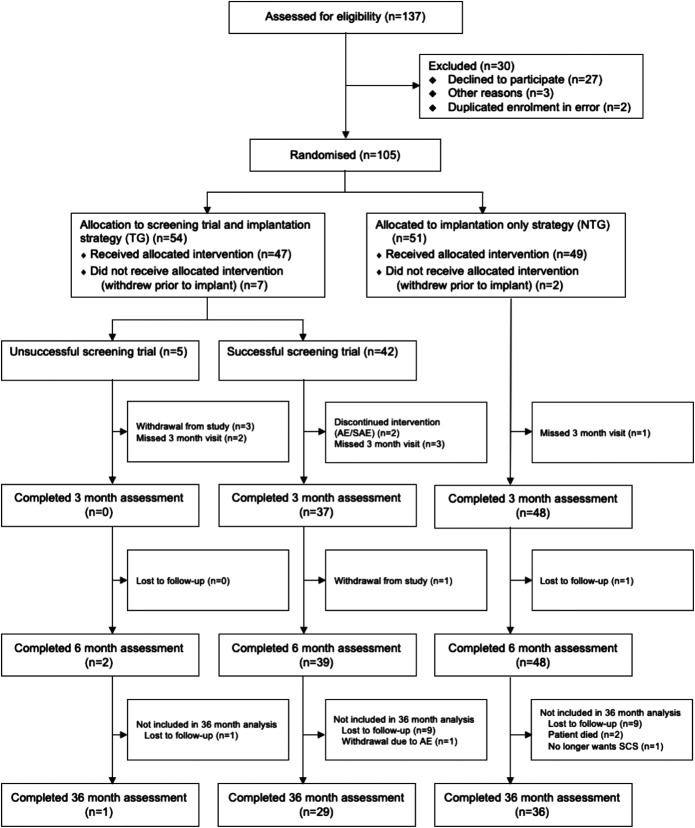
Trial profile. AEs, adverse event; NTG, no trial group; SAE, serious adverse event; TG, trial group.

The only difference between the TG and NTG groups' baseline characteristics and outcome ratings was the duration of pain, which was slightly longer in the NTG group. Participants in the study had a mean NRS pain score of 7.5, an average age of 50.4 years, and fairly equal sex representation. The most frequent primary diagnoses in both groups were PSPS-T2 (52% TG, 55% NTG) and radiculopathy (15% TG, 23% NTG).

At 36-month follow-up, there was no difference in the primary outcome of clinic-assessed NRS score between TG and NTG (adjusted mean difference: −0.60, 95% CI: −1.83 to 0.63; see Table 1). Considering a minimal clinical important difference (MCID) of 2 in the NRS,^35,36^ or recently suggested MCID ranges from 0.9 to 2.7,^37^ there were no clinically relevant differences in pain intensity at 36 months or change from baseline between TG and NTG groups. There was evidence of clinically significant reductions in mean NRS pain from baseline to 36 months for both TG (7.03-4.63) and NTG (7.58-5.47).

**TABLE 1.** Complete Case Analysis of Primary and Secondary Continuous Outcomes

Measure	Time Point	TG (N = 30)		NTG (N = 36)		Unadjusted mean difference (95%CI)^a^	Adjusted mean difference (95%CI)^a,b^
		Mean^a^	SD^a^	Mean^a^	SD^a^		
NRS	Baseline	7.03	1.10	7.58	1.23	−0.55 (−1.12 to 0.02)	–
	36-mo follow-up	4.63	2.48	5.47	2.37	−0.84 (−2.04 to 0.36)	−0.60 (−1.83 to 0.63)
	Change from baseline at 36 mos	−2.40	2.40	−2.11	2.55	−0.29 (−1.54 to 0.96)	−0.23 (−1.47 to 1.00)
EQ-5D	Baseline	0.39	0.20	0.31	0.23	0.08 (−0.02 to 0.19)	–
	36-mo follow-up	0.58	0.20	0.55	0.26	0.03 (−0.08 to 0.15)	−0.02 (−0.13 to 0.10)
	Change from baseline at 36 months	0.19	0.22	0.24	0.31	−0.05 (−0.19 to 0.08)	−0.06 (−0.20 to 0.08)
EQ-VAS	Baseline	48.32	20.10	54.08	21.77	−5.76 (−16.11 to 4.59)	–
	36-mo follow-up	53.87	20.64	59.29	22.79	−5.41 (−16.16 to 5.33)	−4.14 (−14.90 to 6.61)
	Change from baseline at 36 mo	5.55	27.84	5.20	22.94	0.35 (−12.15 to 12.84)	−0.15 (−13.12 to 12.80)

EQ-5D, EuroQol-5 Dimension; EQ-VAS, EuroQol Visual Analog Scale; NRS, numerical rating scale; NTG, no trial group; TG, trial group.

a Means, standard deviation, and unadjusted and adjusted mean difference between groups calculated based on available data (complete case analysis). No imputation of missing data was performed.

b Adjusted for baseline value of the measure (36-month follow-up data only), sex, age (≥65 vs < 65 years), presence of PSPS‐T2 (yes or no), and site.

Improvements were seen in EQ-5D-5L and EuroQol Visual Analog Scale (EQ-VAS) from baseline to 36 months in both groups. There was no evidence of difference between TG and NTG groups for EQ-5D utility scores (adjusted mean difference: −0.02, 95% CI: −0.13 to 0.10). The mean change from baseline at 36-month follow-up for EQ-5D utility scores observed was around 0.2 for both TG and NTG groups. Considering a MCID of 0.074 for the EQ-5D utility scores,^38^ there were no clinically significant differences between the TG and NTG at 36 months or change from baseline. Clinically significant improvements in EQ-5D utility scores were observed at 36 months for both TG (0.19) and NTG (0.24). Similarly, for EQ-VAS, there were no significant differences between TG and NTG groups (adjusted mean difference: −4.14, 95% CI: −14.90 to 6.61).

The proportion of patients who were responders considering a ≥50% (33% TG; 31% NTG) or ≥30% (53% TG; 42% NTG) reduction in pain from baseline were not statistically different between the TG and NTG groups (Table 2).

**TABLE 2.** Responders Per Group According to Level of Pain Reduction (Complete Case Analysis at 36-Month Follow-up)

Measure		TG (N = 30)	NTG (N = 36)	Unadjusted Odds ratio (95%CI)^a^	Adjusted Odds ratio (95% CI)^a,b^	
Reduction in NRS ≥ 50%	Yes: n (%)	10 (33%)	11 (31%)	1.14 (0.35-3.63)	1.33 (0.42-4.23)	
	No: n (%)	20 (67%)	25 (69%)		
Reduction in NRS ≥ 30%	Yes: n (%)	16 (53%)	15 (42%)	1.60 (0.54-4.76)	1.75 (0.61-5.03)	
	No: n (%)	14 (47%)	21 (58%)		

NRS, numerical rating scale; NTG, no trial group; TG, trial group.

a Risk ratio between groups calculated based on available data (complete case analysis). No imputation of missing data was performed.

b Adjusted for baseline value of NRS, sex, age (≥65 vs < 65 years), presence PSPS-T2 of (yes or no), and site.

Considering a MCID of 2 in the NRS,^35,36^ there were no clinically relevant differences in pain intensity at 36 months or change from baseline between TG and NTG groups according to type of SCS programming (Table 3). Similarly, there were no clinically relevant differences according to type of SCS programming when considering the overall trial population.

**TABLE 3.** Pain Intensity Per Group According to SCS Waveform (Complete Case Analysis at 36-Month Follow-up)

SCS Waveform	TG (N = 30)^a^			NTG (n = 36)			Total (n = 66)^a^		
	n	Mean	SD	n	Mean	SD	n	Mean	SD
NRS at 36-month follow-up									
Paresthesia stimulation	11	3.45	2.98	17	4.91	2.12	28	4.34	2.55
HF stimulation	9	5.11	1.36	14	6.11	2.51	23	5.72	2.16
Burst stimulation	9	5.11	2.15	5	5.60	2.79	14	5.29	2.30
Change from baseline in NRS at 36 months									
Paresthesia stimulation	11	−3.18	2.99	17	−2.56	2.73	28	−2.80	2.80
HF stimulation	9	−1.89	2.15	14	−1.50	2.37	23	−1.87	2.18
Burst stimulation	9	−2.44	1.81	5	−2.30	2.59	14	−2.04	2.22

HF, high-frequency; NRS, numerical rating scale; NTG, no trial group; SCS, spinal cord stimulation; TG, trial group.

a Programming method missing for 1 patient in the TG group.

Since the primary 6-month end point, there were 2 explants of the SCS device. One explant in TG because the device was not switching on; a new IPG has been scheduled for implant. There was one explant in NTG because of pain at the IPG site. Between six- and 36-month follow-ups, there were 3 adverse events (AEs) corresponding to fractured leads for 2 patients (1 TG and 1 NTG) and 1 patient in TG reporting problems charging the device; none of these AEs resulted in device explant. Overall (ie, during the 36-month study period, which included 2 additional device explants in TG because of implant-related wound infections), the likelihood of having an explant was not significantly different between TG (3 explants from a total of 54 patients) and NTG groups (1 explant from a total of 51 patients; unadjusted odds ratio 0.34, 95% CI 0.03-3.4). Similarly, no difference was observed for the likelihood of reporting an AE (TG [14 out of 54 patients]; NTG [12 out of 51 patients]; odds ratio 0.88, 95% CI 0.36-2.14).

## DISCUSSION

The results of this study demonstrate that SCS screening trials do not provide superior patient outcomes in the long-term when compared with not doing a screening trial. These findings support the initial conclusions at the 6-month primary end point.^27^ The results further support that the patients' preference to undergo an SCS implant as a single-stage procedure^28^ may not be detrimental to obtain benefits from SCS. It is also noteworthy that at 36- to 40-month follow-up, we observed no difference in the number of explants between the groups.

Clinically important reductions in pain intensity and improvements in health-related quality of life were observed at 36-month follow-up for both groups, confirming the long-term effectiveness of SCS. Previous long-term follow-ups of RCTs have reported similar effects of SCS for patients with neuropathic pain.^39,40^ Although superior to a MCID of 2 points in the NRS,^35,36^ the pain reduction obtained by patients in our study is less than previously reported. RCT samples are usually highly selected with frequent exclusion of participants with a higher risk profile, and therefore, less representative of clinical practice.^41,42^ TRIAL-STIM was a pragmatic RCT, the eligibility criteria used reflects clinical practice in the United Kingdom and is in line with NICE guidance for SCS.^21^ Therefore, we consider that our study provides an overall more realistic representation of the impact of SCS in clinical practice than RCTs that adopt eligibility criteria not reflective of clinical practice. Although it may be argued that the smaller effect of SCS reflects poor patient selection, it is useful to note that a multidisciplinary assessment was part of our inclusion criteria and that this included a psychological assessment at all 3 centers. Furthermore, the low rate of AEs reported in the study compares favorably to other RCTs and supports the expertise of the implanters involved.

Although our study was not powered for a comparison between the 3 waveforms used in this study (paresthesia stimulation, high frequency at 10 kHz, and burst), we did not observe clinically relevant differences between these waveforms at 6 or at 36 months. Discussion considering a smaller MCID is presented in **Supplementary Material 1**, http://links.lww.com/NEU/D381.

Parallel and crossover RCTs have previously reported no differences in effectiveness when comparing different paresthesia-free frequencies,^43^ high frequency (10 kHz) vs paresthesia-inducing stimulation,^44^ high frequency (5 kHz) vs placebo,^45^ and burst vs different frequencies (ie, 40, 500, and 1200 Hz).^46^ However, superior pain reduction has been observed for tonic subthreshold stimulation at 500 Hz vs burst or placebo,^47^ high frequency (10 kHz) vs paresthesia-inducing stimulation,^17^ burst vs tonic stimulation,^16^ or 5882 Hz vs 1200 Hz, 3030 Hz, and sham.^48^ Not only different study settings and population characteristics, but also program settings, number of leads, and electrode contacts, or how sham stimulation was enabled may all contribute to the discrepancy in the results reported. Methodological and reporting deficiencies in trials of SCS have been previously highlighted.^49,50^ Development of SPIRIT and CONSORT extensions specific to implantable neurostimulation devices may potentially lead to improved reporting and transparency of clinical trial protocols and reports while facilitating identification of possible reasons for discrepant findings.^51^

### Strengths

We provide a long-term follow-up of the only RCT to date that evaluated the clinical utility of SCS screening trials. Similar to the TRIAL-STIM RCT, the follow-up assessment was independent from industry funding. Few RCTs of SCS are fully independent of industry and none report results to 36 months. We observed some loss to follow-up; however, this was not substantial, considering that 77% of the participants that completed the primary end point at 6 months completed the follow-up at 36 months.

### Limitations

Analysis of cost-effectiveness at 36 months was not conducted as the length of recall required between assessments (ie, 30 months) would limit interpretation of the results (see **Supplementary Material 2**, http://links.lww.com/NEU/D381). Previously, it had been suggested that the use of screening trials could be cost-saving if at least 20% of patients had an unsuccessful trial.^52^ Recent evidence from a budget impact analysis evaluating the costs or savings of conducting screening trials suggests that this figure may be closer to 14.4%,^53^ an unsuccessful trial failure rate approximately two-fold of previous rates observed in UK clinical practice.^54^

### Practice Implications

Screening trials continue to be recommended by clinical guidelines and required by regulators. The FDA recently issued a letter to healthcare providers reminding of the importance of conducting a screening trial to confirm satisfactory pain relief before implanting a SCS device.^55^ Our findings suggest that the utility of screening trials in identifying long-term responders to SCS may be limited. We consider that patient selection with support from a multidisciplinary team may identify suitable patients who will obtain satisfactory pain relief with SCS in the long term based on pain reduction and limited number of device explants in the NTG group. An e-health tool has been recently developed to assist the assessment of the appropriateness of a patient for referral and selection for SCS, considering clinical and psychosocial factors.^56^ A study of 483 consecutive patients in 12 experienced European centers showed a strong correlation between the retrospective application of the e-tool panel recommendations and SCS trial and treatment outcome.^57^ Further prospective research is ongoing to evaluate the predictive value in e-tool-naive centers that are blinded to the e-tool panel recommendations.

We do not suggest that screening trials should be eradicated, but they should not be made mandatory. An informed decision on whether to perform a screening trial should be based on professional judgment and patient preferences, considering the advantages and disadvantages of screening trials.

## CONCLUSION

The results of this long-term follow-up demonstrate that there is no patient outcome benefit in undertaking a SCS screening trial.

## Supplemental Digital Content

**Supplementary Material 1.** Consideration of a smaller minimally clinical important difference (MCID).**Supplementary Material 2.** Cost-effectiveness analysis considerations.
